# Comparable outcomes with 14-, 21-, or standard 28-day venetoclax in the first cycle of azacitidine–venetoclax in untreated acute myeloid leukemia: real-world experience from the Hokkaido Leukemia Net

**DOI:** 10.1038/s41408-025-01324-7

**Published:** 2025-07-03

**Authors:** Minoru Kanaya, Masahiro Onozawa, Toshihiro Matsukawa, Naoki Miyashita, Fumiaki Fujii, Shota Yoshida, Jun Nagai, Masayuki Aiba, Daisuke Hidaka, Junichi Hashiguchi, Hajime Senjo, Tetsuyuki Igarashi, Masahiro Chiba, Satoshi Yamamoto, Taku Shimizu, Takashi Ishio, Shota Yokoyama, Ko Ebata, Satoshi Iyama, Tatsuo Oyake, Takeshi Kondo, Takanori Teshima

**Affiliations:** 1 Blood Disorders Center, Aiiku Hospital, Sapporo, Hokkaido Japan; 2 Department of Hematology, Hokkaido University Hospital, Sapporo, Hokkaido Japan; 3 Department of Hematology, Hokkaido University Faculty of Medicine, Graduate School of Medicine, Sapporo, Hokkaido Japan; 4 Department of Hematology, Sapporo Hokuyu Hospital, Sapporo, Hokkaido Japan; 5 Department of Internal Medicine, Kitami Red Cross Hospital, Kitami, Hokkaido Japan; 6 Department of Hematology, Kushiro Rosai Hospital, Kushiro, Hokkaido Japan; 7 Department of Hematology, Tenshi Hospital, Sapporo, Hokkaido Japan; 8 Department of Hematology, Asahikawa City Hospital, Asahikawa, Hokkaido Japan; 9 Department of Hematology, Sapporo City General Hospital, Sapporo, Hokkaido Japan; 10 Department of Hematology, Teine Keijinkai Hospital, Sapporo, Hokkaido Japan; 11 Department of Hematology, Sapporo Kosei General Hospital, Sapporo, Hokkaido Japan; 12 Department of Hematology, Obihiro Kosei Hospital, Obihiro, Hokkaido Japan; 13 Department of Hematology, Hokkaido Cancer Center, Sapporo, Hokkaido Japan; 14 Department of Hematology, Sapporo Medical University School of Medicine, Sapporo, Hokkaido Japan; 15 Department of Hematology and Oncology, Iwate Medical University, Yahaba, Iwate Japan

**Keywords:** Acute myeloid leukaemia, Risk factors

Dear Editor,

Acute myeloid leukemia (AML) has a median age at diagnosis of 68 years, and many patients are deemed ineligible for intensive chemotherapy or allogeneic stem cell transplantation, resulting in a generally poor prognosis [1]. The pivotal VIALE-A randomized trial showed that Azacitidine (AZA) at 75 mg/m²/day for 7 days every 28 days, combined with once-daily continuous Venetoclax (VEN) at 400 mg for 28 days, significantly improved composite complete remission (CRc) rates (66.4% vs. 28.3%) and overall survival (OS) (14.7 vs. 9.6 months) compared to AZA alone [2]. The combination of AZA-VEN has become the standard treatment for unfit AML patients, demonstrating high efficacy in real-world settings as well [3]. However, prolonged neutropenia—which increases the risk of febrile neutropenia (FN) and documented infections—remains a major challenge during continuous AZA-VEN therapy. Several approaches have been explored to mitigate this issue, including VEN dose modification, therapeutic drug monitoring [4], and shortening of VEN administration duration [5–8]. Willekens C et al. demonstrated that VEN used for 7 days resulted in similar response rates and survival compared to standard 28 days of VEN exposure. In response to the literature written by Willekens C et al., we investigated the impact of reduced VEN duration for 14 days, 21 days, and 28 days by using a multi-institutional dataset from the Hokkaido Leukemia Net (HLN).

We retrospectively analyzed 100 Japanese patients with untreated AML who were initially treated with AZA-VEN at 14 institutes in the HLN between May 2021 and December 2023 to evaluate VEN duration during the first cycle. In the first cycle, bone marrow aspiration was typically performed on days 14 to 21 of VEN administration. The duration of VEN exposure was determined by the attending physicians and ranged from 11 to 29 days, with a median duration of 21 days. The duration of VEN was divided into three groups: 14 ± 3 days (VEN 14, *n* = 31), 21 ± 3 days (VEN 21, *n* = 51), and 28 ± 3 days (VEN 28, *n* = 18). Efficacy and safety among the three groups (VEN 14, VEN 21, and VEN 28) were evaluated to assess the clinical significance of VEN duration. CRc was defined as CR plus CRi (complete remission with incomplete hematologic recovery). To enable fair comparison of CRc rates across the three groups, we employed our novel risk stratification system for AZA-VEN [9]. Patients were classified as follows: those with NPM1, IDH1/2, or DNMT3A mutations were considered “VEN-sensitive”; those with complex karyotype (CK) or TP53 mutations and without VEN-sensitive mutations were “unfavorable”; all others were “intermediate”. In addition to CRc rate, OS, frequency of FN, grade 4 neutropenia (less than 500/µl), and occurrence of documented infection in the 1st cycle were assessed. VEN dosage was adjusted to accommodate azole antifungal prophylaxis. The patient’s characteristics were tested by kai-square and Fisher’s exact tests for categorical data or the t-test for quantitative data. OS was estimated from the time of diagnosis to last follow-up or death and evaluated by the Kaplan–Meier method with differences compared by the log-rank test. Statistical analysis was performed using GraphPad Prism ver 10.4.2. This study was part of a prospective observational study (HLN, UMIN000048611). It was conducted in compliance with ethical principles based on the Helsinki Declaration and was approved by the institutional review board of Hokkaido University Hospital (#015- 0344).

The median age of the cohort was 74 years (range: 47–91), and the median follow-up duration was 358 days (range: 13–1291). AZA was administered at 75 mg/m^2^ from day 1 to 7. The median VEN dose was 200 mg (range: 50–400 mg). Patient characteristics for the three groups (VEN 14, VEN 21, and VEN 28) are summarized in the Table 1. Baseline characteristics—such as age, sex, and follow-up duration—were similar among groups. Disease profiles, including the incidence of secondary AML, cytogenetic risk, representative somatic mutations (FLT3-ITD, NPM1, IDH1/2, N/KRAS, TP53), and the HLN risk classification, were well balanced. The proportions of azole prophylaxis and G-CSF use, as supportive measures, were also comparable across groups. Notably, the median VEN dose was relatively lower in the VEN 14 group. As shown in Table 1, CRc rates for VEN 14, VEN 21, and VEN 28 were 67.7%, 51.0%, and 38.9%, respectively, with no statistically significant differences. According to the HLN risk stratification system, CRc rates in the VEN-sensitive group were 83.3% (10/12), 71.4% (10/14), and 83.3% (5/6) in VEN 14/21/28, respectively. CRc rates in the intermediate risk group for VEN 14/21/28 were 61.5% (8/13), 45.6% (10/22), and 25.0% (1/4), respectively. In the unfavorable group, CRc rates were 50.0% (3/6), 40.0% (6/15), and 12.5% (1/8). No statistical significance of CRc rate was observed among VEN 14/21/28, stratified by the HLN risk classification. OS did not significantly differ among VEN 14, VEN 21, and VEN 28 groups: 481, 438, and 240 days, respectively (Fig. 1). Time from initiation of the first cycle to the start of the second cycle reflecting the duration of the first cycle was assessed as 43 days (range: 29–54) in VEN 14, 46 days (range: 28–60) in VEN 21, and 36 days in VEN 28 (28–91), with no statistically significant (Supplementary Fig. 1). In terms of the safety profile, the frequency of grade 4 neutropenia, FN, and documented infection in VEN 14/21/28 were as follows: grade 4 neutropenia—67.7%, 78.4%, and 50.0%; FN—54.8%, 47.1%, and 38.9%; documented infection—25.8%, 11.7%, and 16.7%. These adverse events did not differ significantly among the three groups. The median duration of grade 4 neutropenia showed no significant differences among the groups: 29 days (range: 21–57) in VEN 14, 32 days (range: 24–52) in VEN 21, and 33 days (range: 20–63) in VEN 28. The duration of FN was also similar between the 3 groups: 5 days (range: 1–15) in VEN 14, 4 days (range: 1–42) in VEN 21, and 7 days (range: 2–25) in VEN 28.

**Table 1:** Patient characteristics and treatment outcome across VEN14, VEN21, and VEN28.

	VEN 14 (*n* = 31)	VEN 21 (*n* = 51)	VEN 28 (*n* = 18)	*p*-value
Median age, year (range)	79 (63–87)	71 (61–88)	76 (63–91)	0.708
Male *n* (%)	19 (61.3%)	29 (56.9%)	8 (44.4%)	0.511
Median follow-up	456 days (13–1141)	384 days (19–1291)	226 days (37–1155)	
Secondary AML	7 (22.6%)	13 (25.5%)	3 (16.7%)	0.745
Prior MDS	4 (12.9%)	12 (23.5%)	0 (0.0%)	0.079
Therapy-related AML	3 (9.7%)	1 (2.0%)	3 (1.7%)	0.054
Cytogenetics				0.100
Normal	15 (19.4%)	19 (37.3%)	6 (33.3%)	
Complex	2 (6.5%)	13 (25.5%)	7 (38.9%)	
Others	14 (45.2%)	19 (37.3%)	5 (27.8%)	
Mutaions				
FLT3-ITD	5 (16.1%)	2 (3.9%)	2 (11.1%)	0.150
NPM-1	3 (9.7%)	4 (7.8%)	5 (27.8%)	0.086
IDH1/IDH2	9 (29.0%)	12 (23.5%)	5 (27.8%)	0.844
N/KRAS	0 (0.0%)	2 (3.9%)	0 (0.0%)	0.681
TP53	9 (29.0%)	14 (27.5%)	7 (38.9%)	0.654
HLN risk stratification for AZA-VEN				0.304
Sensitive (*n* = 32)	12 (38.7%)	14 (27.5%)	6 (33.3%)	
Intermediate (*n* = 39)	13 (41.9%)	22 (43.1%)	4 (22.2%)	
Unfavorable (*n* = 29)	6 (19.4%)	15 (29.4%)	8 (44.4%)	
Azole prophylaxis in 1st cycle	7 (22.6%)	19 (37.3%)	3 (16.7%)	0.162
G-CSF in 1st cycle	12 (38.7%)	23 (45.1%)	5 (27.8%)	0.429
Median VEN dose (range)	200 mg (50–400)	400 mg (50–400)	400 mg (50–400)	0.021
CRc rate(%)				
All patients	21 (67.7%)	26 (51.0%)	7 (38.9%)	0.123
VEN-sensitive (*n* = 32)	10 (83.3%)	10 (71.4%)	5 (83.3%)	0.857
VEN-intermediate (*n* = 39)	8 (61.5%)	10 (45.6%)	1 (25.0%)	0.369
VEN-unfavorable (*n* = 29)	3 (50.0%)	6 (40.0%)	1 (12.5%)	0.336
Grade 4 neutropenia (%)	21 (67.7%)	40 (78.4%)	9 (50.0%)	0.073
Febrile neutropenia (%)	17 (54.8%)	24 (47.1%)	7 (38.9%)	0.550
Documented infection (%)	8 (25.8%)	6 (11.7%)	3 (16.7%)	0.260
Duration of grade 4 neutropenia (days)	29 days (21–57)	32 days (24–52)	33 days (20–63)	0.731
Duration of febrile neutropenia (days)	5 days (1–15)	4 days (1–42)	7 days (2–25)	0.890
Median OS (days)	481 days	438 days	240 days	0.210

**Fig. 1:**
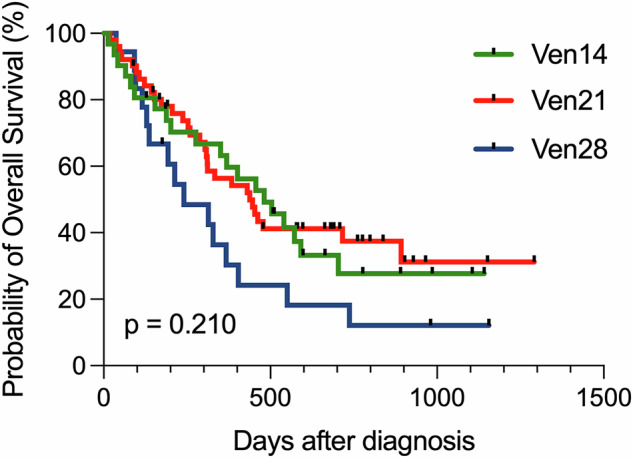
Overall survival of “VEN 14” vs. “VEN 21” vs. “VEN 28”. Overall survival stratified by venetoclax duration, 14 days administration of venetoclax (VEN 14), 21 days administration of venetoclax (VEN 21), and 28 days of venetoclax (VEN 28).

According to ELN recommendations [10], shortening VEN administration from 28 to 21 or even 14 days is generally allowed during the consolidation phase to prevent delayed neutrophil recovery. In this study, we analyzed real-world data on shortened VEN duration during the induction phase. Consistent with previous reports from the Mayo Clinic [5] and Willekens et al. [7], shortening VEN duration during the first cycle of AZA-VEN in newly diagnosed AML patients from the HLN cohort did not impact outcomes such as CRc rate, OS, documented infections, or the frequency and duration of grade 4 neutropenia or FN. Our findings suggest that shorter VEN durations (14 or 21 days) are both effective and safe in an East Asian population. Although not statistically significant, the VEN 14 and VEN 21 demonstrated a trend toward longer survival compared to the VEN 28 group, which included slightly higher proportion of patients harboring TP53 mutation. We further compared outcomes among high-risk patients with CK/TP53 mutations, the VEN 14 and VEN 21 groups exhibited numerically higher CRc rates than the conventional VEN 28 group, though not statistically significant—suggesting potential effectiveness of shorter VEN durations even in high-risk patients. Willekens et al. [7] reported that limiting VEN to 7 days reduced OS compared to the standard 28-day duration in patients predicted to benefit from VEN based on the molecular prognostic risk signature (mPRS). In contrast, our data showed that VEN 14 and VEN 21 did not compromise treatment efficacy, even among patients with favorable genetic mutations.

This study had several limitations. First, the cohort size was insufficient to definitively establish the non-inferiority of shortened VEN durations compared to the standard 28-day regimen. Second, since our HLN AZA-VEN cohort was retrospective, patient backgrounds across the three VEN duration groups (VEN 14, VEN 21, and VEN 28) were not entirely comparable. The physician’s intention to select VEN duration could exist. The range of VEN duration was relatively varied because the study was in a real-world setting. We evaluated that the duration of VEN wasn’t linearly correlated with the duration of neutropenia and FN (data not shown). Based on these data, we decided that a difference of ±3 days has no apparent clinical significance. Finally, we didn’t stratify disease risk by mPRS and Mayo Genetic Risk Models, commonly used [11–14]. Our original HLN risk stratification should work well in the HLN cohort.

In summary, our extensive analysis demonstrates the feasibility and potential benefits of 14 days VEN and 21 days administration in a real-world setting. Notably, the efficacy of shorter VEN administration remained consistent even when stratified according to the AZA–VEN-specific risk classification developed by the HLN. These findings contribute to ongoing discussions regarding the optimal VEN duration and may help guide personalized treatment strategies. The results of an ongoing prospective study comparing VEN 14 versus VEN 28 (NCT03013998) [15] are eagerly awaited. A novel AZA-VEN plus a new drug combination regimen is developing at present, and it is important to build new evidence to evaluate the proper duration of VEN.

## Supplementary information

Supplemental Material

## Data Availability

The datasets for the cohort study generated during and/or analyzed during the current study are available from the corresponding author upon reasonable request.
