# Supplementary material for: Comparable outcomes with 14-, 21-, or standard 28-day venetoclax in the first cycle of azacitidine–venetoclax in untreated acute myeloid leukemia: real-world experience from the Hokkaido Leukemia Net
Source: Blood Cancer J. 2025 Jul 3;15(1):118. doi: 10.1038/s41408-025-01324-7 (PMC12229622; doi:10.1038/s41408-025-01324-7)
Supplement: Supplementary file 1 — Supplemental Material [file 41408_2025_1324_MOESM1_ESM.docx]

**
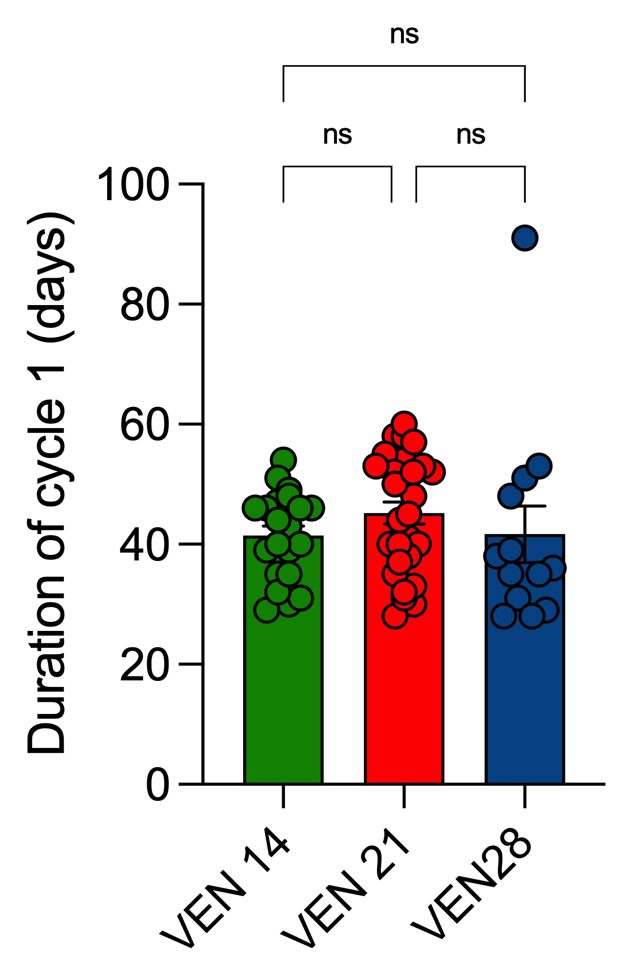
**

**Figure S1. Duration of cycle 1**

Duration of cycle 1 was evaluated among 3 groups, 14 days administration of VEN, 21 days administration of VEN, and 28 days of administration of VEN. (ns. not significant, t-test)
